# Photodynamic Therapy for Head and Neck Cancer

**DOI:** 10.1155/DTE.3.41

**Published:** 1996

**Authors:** Tomoyuki Yoshida, Harubumi Kato, Tetsuya Okunaka, Tetsuo Saeki, Shinya Ohashi, Tadao Okudaira, A. Masaji Lee, Hikari Yoshida, Hidehiro Maruoka, Hiroyuki Ito, Sotaro Funasaka

**Affiliations:** 1 Department of Otolaryngology, Head and Neck Surgery Tokyo Medical College 6-7-1 Nishishinjuku, Shinjuku-ku Tokyo 160 Japan; 2 Department of Surgery Tokyo Medical College 6-7-1 Nishishinjuku, Shinjuku-ku Tokyo 160 Japan

## Abstract

Photodynamic therapy (PDT) is a recently developed treatment involving the use of a photosensitizer and low power light, usually from a laser, to selectively destroy tumor cells. At present, we perform PDT for head and neck cancer using argon or excimer dye lasers with hematoporphyrin derivative as a photosensitizer. This study attempted to assess the utility and safety of PDT and to investigate the long-term outcome. All 24 patients had squamous cell carcinoma: 15 with laryngeal, 5 with lingual or oral, and 4 with pharyngeal cancer and were treated by PDT. Data were obtained from records from February 1988 through April 1995. After PDT, 12 of 15 laryngeal cancer patients were classified as having a complete remission (CR), as were 2 of the 5 lingual or oral and one of the 4 pharyngeal cancer patients. The patients were followed for 8 to 153 months. The longest duration of CR in patients treated by PDT alone was 148 months. Photosensitivity was experienced by all patients, but required no treatment. Liver, kidneys, and bone marrow showed no abnormal values. There were no clinically relevant adverse reactions, and patients with severe complications due to other types of treatment and elderly patients were also treated safely with this therapy.


*Diagnostic and Therapeutic Endoscopy*, Vol. 3, pp. 41–51
Reprints available directly from the publisher
Photocopying permitted by license only

© 1996 OPA (Overseas Publishers Association)
Amsterdam B.V. Published in The Netherlands
by Harwood Academic Publishers GmbH
Printed in Singapore

### Photodynamic Therapy for Head and Neck Cancer

TOMOYUKI YOSHIDA*, HARUBUMI KATO, TETSUYA OKUNAKA, TETSUO SAEKI, SHINYA OHASHI, TADAO OKUDAIRA, A. MASAJI LEE, HIKARI YOSHIDA, HIDEHIRO MARUOKA, HIROYUKI ITO and SOTARO FUNASAKA

*Departments of Otolaryngology, Head and Neck Surgery, (T.Y., T.S., S.O., T.O., A.M.L., H.Y., H.M., H.I., S.F.), and Surgery (H.K., T.O.), Tokyo Medical College, 6-7-1 Nishishinjuku, Shinjuku-ku, Tokyo 160, Japan*

*(Received 12 December 1995; In final form 20 March 1996)*

**Photodynamic therapy (PDT) is a recently developed treatment involving the use of a photosensitizer and low power light, usually from a laser, to selectively destroy tumor cells. At present, we perform PDT for head and neck cancer using argon or excimer dye lasers with hematoporphyrin derivative as a photosensitizer. This study attempted to assess the utility and safety of PDT and to investigate the long-term outcome. All 24 patients had squamous cell carcinoma: 15 with laryngeal, 5 with lingual or oral, and 4 with pharyngeal cancer and were treated by PDT. Data were obtained from records from February 1988 through April 1995. After PDT, 12 of 15 laryngeal cancer patients were classified as having a complete remission (CR), as were 2 of the 5 lingual or oral and one of the 4 pharyngeal cancer patients. The patients were followed for 8 to 153 months. The longest duration of CR in patients treated by PDT alone was 148 months. Photosensitivity was experienced by all patients, but required no treatment. Liver, kidneys, and bone marrow showed no abnormal values. There were no clinically relevant adverse reactions, and patients with severe complications due to other types of treatment and elderly patients were also treated safely with this therapy.**

*Keywords:* Photodynamic therapy, head and neck cancer, low power dye laser, hematoporphyrin derivative

### INTRODUCTION

Since the early 1900s it has been known that photosensitive substances can induce photochemical reactions when exposed to light [1], and this phenomenon suggested that a therapeutically beneficial photochemical reaction can be induced in malignant tumors by tumor-specific photosensitizers. Recently, attention has been focused on photodynamic therapy (PDT) for cancer, using tumor-specific photosensitizers and low-energy laser light. Since Dougherty *et al.* [2] reported the first application of PDT for cancer treatment in 1978, this

*Corresponding author. Tel.: 81-3-3342-6111. Fax: 81-3-3346-9275.

procedure has become increasingly accepted, especially in lung cancer [3] and cervical carcinoma. It has also been applied in patients with cancer of the stomach, esophagus, and bladder. The widening of indications is related to the development of tumor-specific photosensitizers, such as hematoporphyrin derivative (HpD), which have low toxicity and mutagenicity but good affinity to tumors and also the development of low-energy laser light sources and endoscopes to transmit laser light to target lesions. Concerning application to head and neck cancer, several reports have been published from the United States and Europe on laryngeal papilloma [4] recurrent tumor [5], and oral cancer, while in Japan, there has only been one report on laryngeal carcinoma by Yoshida *et al.* [6]. We reviewed the efficacy, safety, and long-term outcome of PDT in patients with head and neck cancer.

### APPLICATION OF PHOTODYNAMIC THERAPY

### Principle

When photosensitizers are excited by absorbing light energy, a physicochemical reaction will occur by transition of energy state. In this reaction, the excited photosensitizers emit energy, activate oxygen in tissue, and produce activated oxygen. Since activated oxygen has cytotoxic effects, degeneration and destruction of tissue cells may occur (Fig. 1).

### Photosensitizers and Laser System

To obtain an adequate photodynamic effect, a hematoporphyrin hydrochloride derivative, HpD, is used as a photosensitizer because it has sufficient affinity to tumors and low toxicity against normal tissue [7,8]. Thus, selective therapy is capable by irradiating the laser beam during the period when HpD is accumulated in tumoral tissue [9]. The HpD can be excited by relatively highly histopermeable red light (630 nm) generated by an argon dye laser. However, because the generated laser light is a continuous sinusoidal wave, the histopermeability and the amount of photons are limited. To resolve this problem, an excimer dye laser, which can generate a pulsed beam, has been applied [10–12]. This device can generate a peak output sufficiently high to produce a transient high-density excitation state and improve the histopermeability. We used two devices, an argon dye laser and a excimer dye laser, for treatment of head and neck cancers.

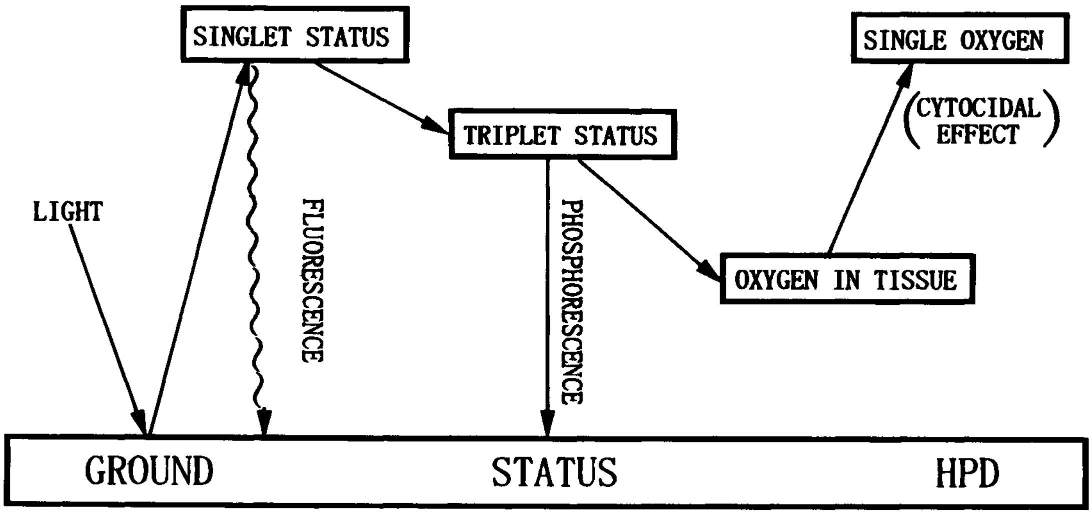

FIGURE 1 Principle of PDT.

### SUBJECTS AND METHODS

### Subjects

The subjects were 24 patients with squamous cell carcinoma who underwent PDT from February 1988 through April 1995. The mean age was 65 years (range, 45 to 86 years). Of 15 patients with laryngeal carcinoma, 13 patients underwent PDT as the first treatment of choice (T 1a: 6 patients; T 1b: 3 patients; T2: 3 patients; and T3: 1 patient), and 2 patients were treated for recurrence after radiotherapy. PDT was the only possible choice in 2 patients with T2 cancer, 3 patients with T3 cancer who refused other therapeutic modalities, and also in 2 patients with recurrence after radiotherapy, one who refused total laryngectomy and one who had synchronous combined cancer. PDT with the argon dye laser was used for initial treatment in 3 patients and for treatment of recurrence in 1 patient, and the excimer dye laser was applied for all other patients. In all 5 lingual or oral cancer patients the lesions were less than 4 cm in maximum dimension. Four of the patients were treated by the argon dye laser, while the last one was treated with an excimer dye laser. In the 4 patients with pharyngeal carcinoma, PDT was included in a multidisciplinary therapeutic regimen in 2 patients and was performed for treatment of locally recurrent lesions after chemotherapy and radiotherapy in 1 patient (Table I). Informed consent was obtained from all patients.

### Methods

HpD (2.0 mg/kg) was administered intravenously 48 hr before the laser irradiation. In 5 patients (cases 1 to 4 and 14), laser light transmitted by a quartz fiber (400 μm) was applied to the lesion under general anesthesia during direct endoscopic observation.

TABLE I PDT for Head and Neck Cancer

| Case No. | Age (yr) | Sex | T | Laser:En/Joule | Effects of PDT |
|---|---|---|---|---|---|
| Larynx | | | | | |
| 1 | 73 | M | T1a | Ex: 180 | CR |
| 2 | 69 | M | T1a | Ar:300;Ex:150 | CR |
| 3 | 67 | M | T1a | Ar:1056 | CR |
| 4 | 66 | M | T1a | Ex:200;Ex:100 | CR |
| 5 | 53 | M | T1a | Ex:100 | CR |
| 6 | 68 | M | T1a | Ex:200;Ex:200 | CR |
| 7 | 70 | M | T1b | Ex:200;Ex:200 | CR |
| 8 | 79 | M | T1b | Ex:200 | PR |
| 9 | 67 | M | T1b | Ex:200 | CR |
| 10 | 72 | M | T2 | Ar:500;Ar:500 | PR |
| 11 | 68 | M | T2 | Ex:650 | PR |
| 12 | 62 | M | T2 | Ex:200 | CR |
| 13 | 66 | M | T3 | Ex:200 | CR |
| 14 | 63 | M | rT1 | Ar:810 | CR |
| 15 | 45 | M | rT2 | Ar:800 | CR |
| Lingual or oral | | | | | |
| 16 | 67 | M | T2 | Ar:500 | PR |
| 17 | 56 | F | T1 | Ar:300 | PR |
| 18 | 76 | M | T1 | Ar:300 | PR |
| 19 | 51 | M | T1 | Ar:500 | CR |
| 20 | 51 | M | T1 | Ex:200 | CR |
| Pharynx | | | | | |
| 21 | 54 | M | T2 | Ar:450;Ar:300 | PR |
| 22 | 65 | M | T3 | Ar:500;Ar:1100 | PR |
| 23 | 69 | F | rT2 | Ar:250 | PR |
| 24 | 86 | M | T3 | Ex:400 | CR |

r, retreated patients; Ex, excimer; Ar, argon.

Other patients underwent a more sophisticated procedure; i.e., under local anesthesia after insertion of a videoendoscope, a quartz fiber was inserted via the biopsy channel and PDT was performed. With the argon dye laser (Fuji Photo Optical Co., Ltd.) 200 to 500 mW/cm$^2$ was delivered by continuous wave laser light for 20 min, while the excimer laser (Hamamatsu Photonics Co.) delivered 3 to 4 mJ/pulse (30 Hz), 100 to 150 J/cm$^2$ or 3 to 4 mJ/pulse (40 Hz), 200 J/cm$^2$. The energy dose was calculated according to the following equation;

$$En = 200\text{–}500\text{mW} \times \text{irradiation time (sec)/cm}^2$$
(argon dye laser)

$$En = (3\text{–}4\ \text{mJ/pulse} \times 30{,}40\ \text{ or } 80\ \text{Hz/sec})/1000 \times \text{irradiation time (sec)/cm}^2$$

(excimer dye laser)

We set the basic dose at an energy level of 200 J/cm$^2$ (4 mJ $\times$ 40 Hz $\times$ 60 sec $\times$ 21 min/1000 $\fallingdotseq$ 200 J/cm$^2$) (Table II).

Since a long period of radiation was difficult under local anesthesia due to occasional swallowing and cough reflex, high dose irradiation using laser light with 80 Hz was attempted to reduce the irradiation time. However, this procedure does not seem to be suitable for routine therapy because moderate flare and swelling sometimes occur in the normal mucosa. In the larynx, superficial irradiation is effective in cancers on the surface of the glottis, while quartz fibers with perpendicular lens or a cylindrical quartz fibers are required for cancers in the lower surface or border of the larynx. In addition, photoradiation must be performed with the tip of the transmitting fiber at a distance of 1 cm from the target surface to prevent the defocus phenomenon in all cases.

TABLE II PDT Procedure

| |
|---|
| Intravenous injection of hematoporphyrin derivative (2.0 mg/kg) 48 hr before laser irradiation |
| Laser-power<br>Argon dye laser: continuous wave 200–500 mW/cm2<br>Excimer dye laser: pulse 3– 4 mJ/P (30 Hz) |
| Optimal dose of laser energy: 200 J/cm$^2$ |
| Optimal wave length: 630 nm |

### RESULTS

### Effects of Primary Therapy of PDT

Among 13 patients with laryngeal cancer who underwent PDT as the first treatment of choice; 1 patient with T1b and 2 patients with T2 resulted in partial remission (PR), while the other 10 patients achieved complete remission (CR), resulting in a CR rate of 76.9%. In 9 T1 patients, a CR was recognized in 8 and a PR was recognized in 1 who refused repeated PDT. In addition, 2 patients with T2 cancer who underwent PDT for recurrent lesions initially treated by radiotherapy regained a CR for as long as 5 and 13 months, and 1 T3 patients also attained a CR. Consequently the overall CR rate was 80.0%.

In 5 patients with lingual or oral squamous cell carcinoma, residual cancer was detected in all resected specimens of the argon dye laser group except for patient 19. In patient (case 20), the first patient to undergo excimer dye laser irradiation for lingual cancer, a CR was obtained. The argon dye laser was used in 4 patients with pharyngeal cancer, 2 of whom were treated with PDT as part of a multidisciplinary regimen (cases 21 and 22) and 1 was treated for local recurrence after chemotherapy and radiotherapy (case 23). In all those patients a PR was achieved in spite of manifestation of a severe cell necrotic reaction. On the other hand a CR was achieved in 1 patient (case 24) with T3 cancer by excimer dye laser irradiation alone.

### Effective Period and Long-Term Results of PDT

The longest follow-up period was 153 months. The first 2 patients were not evaluated because they underwent prophylactic radiotherapy after 2 months. In other patients with laryngeal carcinoma, a CR after PDT alone was maintained for 148 months. In patients treated with PDT as the initial treatment, all cancers were locally controlled except for 1 patient

(T1a) who underwent radiotherapy for local recurrent cancer after 21 months. One patient (case 14) with a T3 lesion of the larynx is at present disease-free after 44 months. All patients with recurrence after radiotherapy had to undergo laryngectomy for cure. Our single patient with recurrence after PDT was successfully treated by radiotherapy. The larynx preservation rate was 85.7%, and in all patients who underwent PDT as a primary therapy the larynx has been preserved. One patient with lingual and one with oral cancer attained CR and are free of disease after 148 months. PDT was performed as one arm of multidisciplinary therapy in most patients with pharyngeal cancers, but a CR was obtained in only 1 lesion, which did not recur for 15 months in spite of it having been T3. Two patients treated for laryngeal cancer died within 24 months from leukemia and esophageal cancer, respectively. Our patient treated for lingual cancer died from esophageal cancer. Two patients with pharyngeal carcinoma died from their disease (Table III).

### Adverse Effects and Complications of PDT

Skin photosensitization occurred in all patients, but no specific treatment was required. Patients were advised not to go out during the daytime, to avoid sunlight, and to apply sunscreen cream for a few weeks after HpD administration [13]. Laboratory test results indicated that liver, kidneys, and bone marrow functions were not affected by PDT. One patient with laryngeal carcinoma underwent tracheotomy due to dyspnea caused by laryngeal edema and glottic fur. In most patients with laryngeal carcinoma, it was necessary to remove fur endoscopically and perform tracheal lavage several times. Severe edema occurred in patients with lin-

TABLE III Effective Period and Outcome of PDT for Head and Neck Cancer

| Case No. | T | Effective Period | After Treatment | Outcome |
|---|---|---|---|---|
| Larynx | | | | |
| 1 | T1a | CR, 2 mo | Prophylactic irradiation | DOA |
| 2 | T1a | CR, 67 mo | | NED |
| 3 | T1a | CR, 2 mo | Prophylactic irradiation | NED |
| 4 | T1a | CR, 21 mo | Radiation for local recurrence | NED |
| 5 | T1a | CR, 61 mo | | NED |
| 6 | T1a | CR, 17 mo | | NED |
| 7 | T1b | CR, 17 mo | | NED |
| 8 | T1b | PR | Radiation | NED |
| 9 | T1b | CR, 8 mo | | NED |
| 10 | T2 | PR | Partial larnygectomy | NED |
| 11 | T2 | PR | | Unknown |
| 12 | T2 | CR, 8 mo | | NED |
| 13 | T3 | CR, 44 mo | | NED |
| 14 | rT1 | CR, 5 mo | Total laryngectomy | NED |
| 15 | rT2 | CR, 13 mo | Total laryngectomy | DOA |
| Lingual or oral | | | | |
| 16 | T2 | PR | Hemiglossectomy | NED |
| 17 | T1 | PR | Partial glossectomy | NED |
| 18 | T1 | PR | Partial glossectomy | DOA |
| 19 | T1 | CR, 148 mo | | NED |
| 20 | T1 | CR, 65 mo | | NED |
| Pharynx | | | | |
| 21 | T2 | PR | Radiation | NED |
| 22 | T3 | PR | Radiation | DOD |
| 23 | rT2 | PR | Radiation | DOD |
| 24 | T3 | CR, 15 mo | | NED |

DOA, death of another; NED, no evidence of disease; DOD, death of disease.

gual or oral cancer; however, most patients could eat without any problem. In 1 patient (case 24), however, feeding via a nasogastric tube for 2 weeks was necessary due to extensive erosive ulcers in the oral cavity. Table I summarizes the results for all patients. The following case reports are representative.

### *Case 1*

A patient with T1a laryngeal cancer was treated by excimer dye laser radiation at 180 J/cm$^2$ under general anesthesia. Because this was the first patient and there was a risk of dyspnea due to severe local reaction, the patient underwent simultaneous prophylactic tracheotomy. During this period, mucosal edema and accumulation of necrotic tissue reduced the laryngeal lumen, requiring frequent endoscopic removal of necrotic tissue. Cytological diagnosis during the observation period revealed Class III b cells although no recurrent cancer was detected macroscopically; therefore, prophylactic 60 Gy Lineac irradiation was given. Figure 2 shows the pretreatment appearance (upper) and appearance 4 days after PDT (middle) and 4 months after PDT (lower). The tracheostomy was closed after 1 week, and no recurrent cancer was detected for 24 months, but the patient died of leukemia.

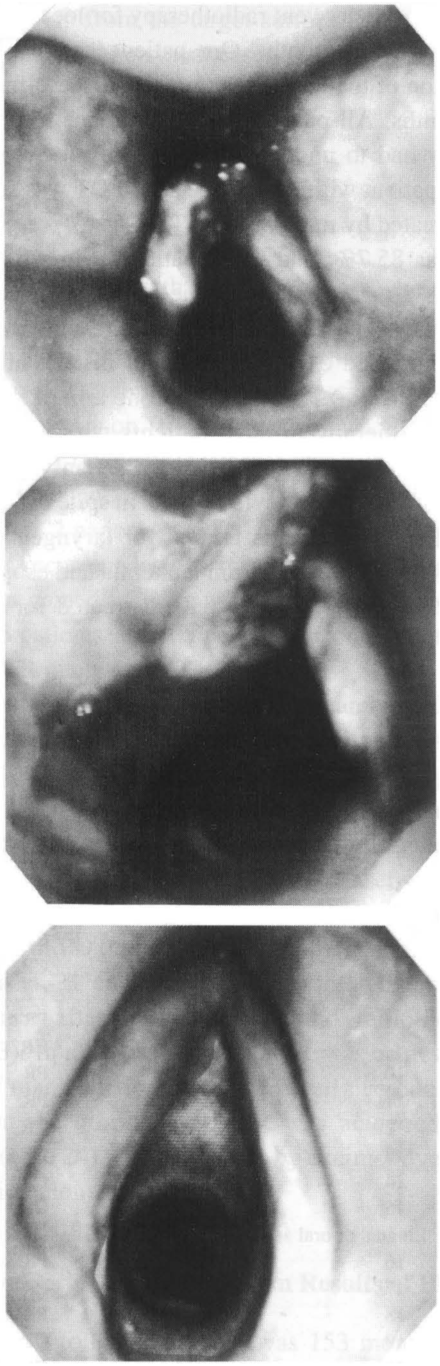

FIGURE 2 This patient (case 1) had T1a laryngeal cancer and was treated by excimer dye laser radiation at 180 J/cm2 under general anesthesia. Necrotic tissue was accumulated, and the laryngeal mucosa became edematous. Prophylactic irradiation was performed with 60 Gy of lineac electron radiation. Top, pretreatment view; middle, 4 days after PDT; bottom, 4 months after PDT. No recurrent cancer was detected for 24 months.

### *Case 7*

In a patient with T1b laryngeal cancer, PDT with an excimer dye laser at 200 J/cm$^2$ of energy was performed twice under local anesthesia and observation by a videoendoscope. Insufficient results were obtained by the first PDT so a repeat procedure was performed (Fig. 3). No recurrence was observed after the second therapy.

### *Case 13*

A patient with T3 laryngeal cancer who refused radiotherapy or surgery was transferred to our hospital. Under local anesthesia 200 J/cm$^2$ from an excimer dye laser was given under videoendoscopic observation. Although we doubted the efficacy of PDT in this T3 patient, a CR was obtained after a single PDT session, and no recurrent cancer has occurred for 42 months (Fig. 4).

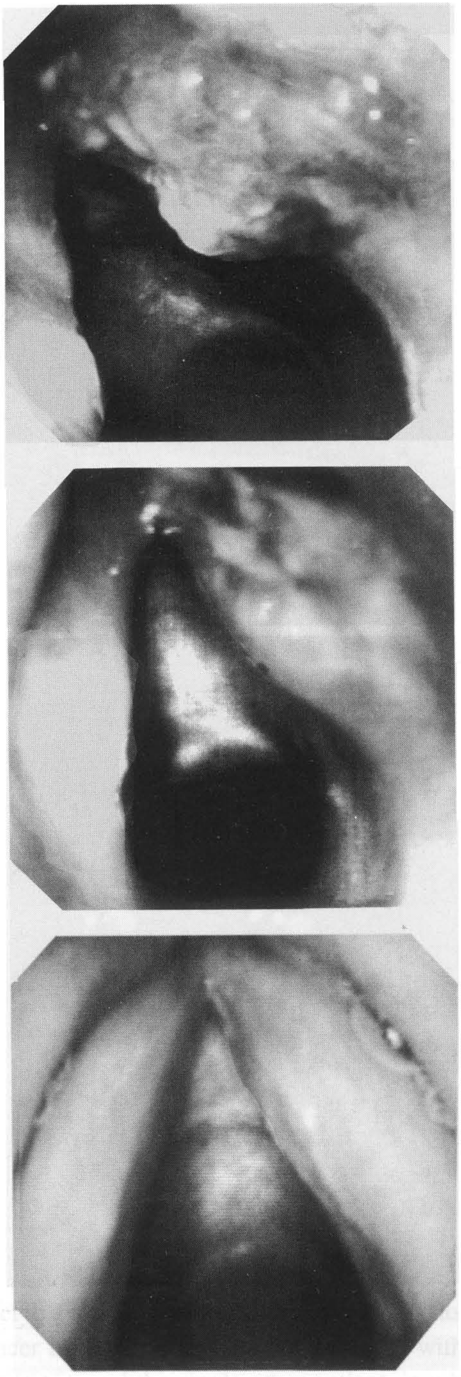

FIGURE 3 This patient (case 7) suffered from T1b laryngeal cancer and underwent PDT with an excimer dye laser at 200 J/cm$^2$ twice under local anesthesia, during observation by videoendoscopy. Top, pretreatment view; middle, 4 days after PDT; bottom 10 months after PDT.

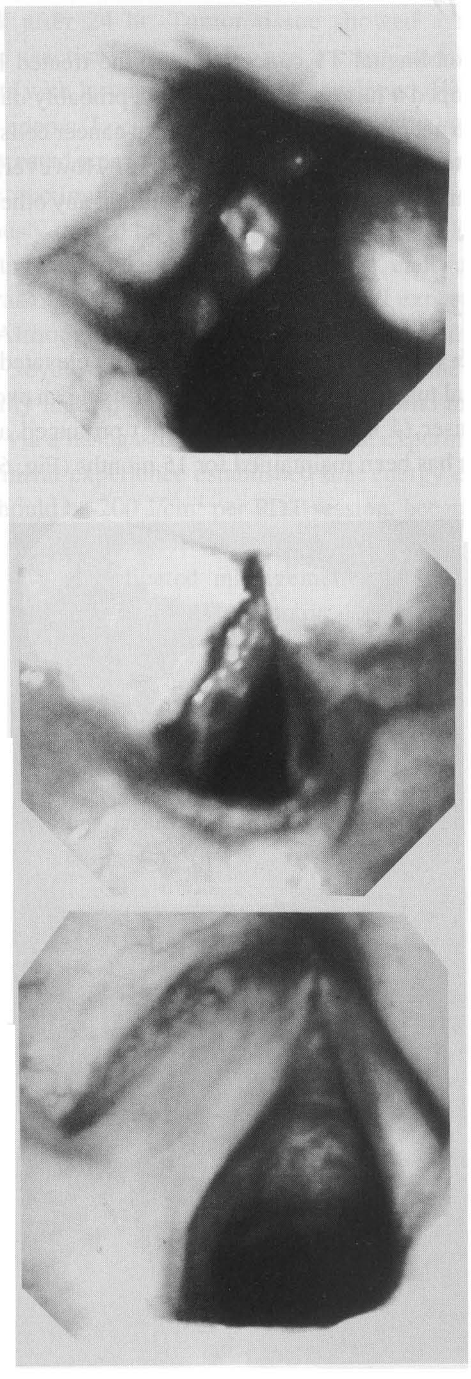

FIGURE 4 This patient (case 10) suffered from T3 laryngeal cancer, but refused radiotherapy or surgery. Under local anesthesia, 200 J/cm$^2$ of energy was radiated with an excimer dye laser. A CR was attained by the first irradiation treatment, and no recurrence has occurred at 42 months. Top, pretreatment view; middle, 4 days after PDT; bottom, after 30 months.

### *Case 17*

In a sublingual T1 cancer patient, the treated lesion developed a furry surface after PDT probably due to a severe necrotic reaction, but residual cancer cells were detected in the resected specimen. However, this patient is alive after 153 months without any other particular treatment (Fig. 5).

### *Case 24*

An 86-year-old man had a T3 markedly elevated pharyngeal tumor in the soft palate. PDT using an excimer dye laser (4 mJ, 30 Hz, 400J/cm$^2$) produced a CR, which has been maintained for 15 months (Fig. 6).

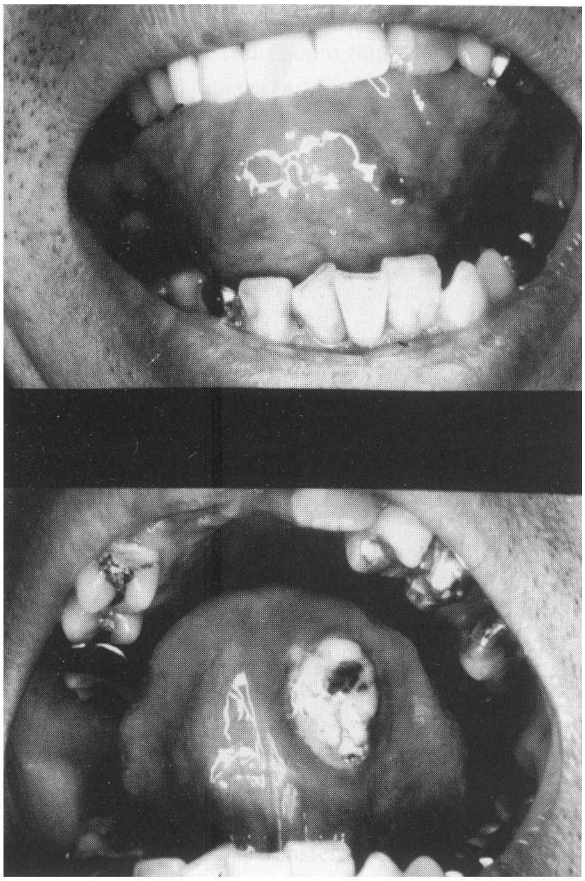

FIGURE 5 This patient (case 17) suffered from sublingual T1 cancer. Although the treated lesion showed fur after PDT, probably due to a severe necrotic reaction, residual cancer cells were detected in the resected specimen. Top, pretreatment view; bottom, 2 weeks after PDT.

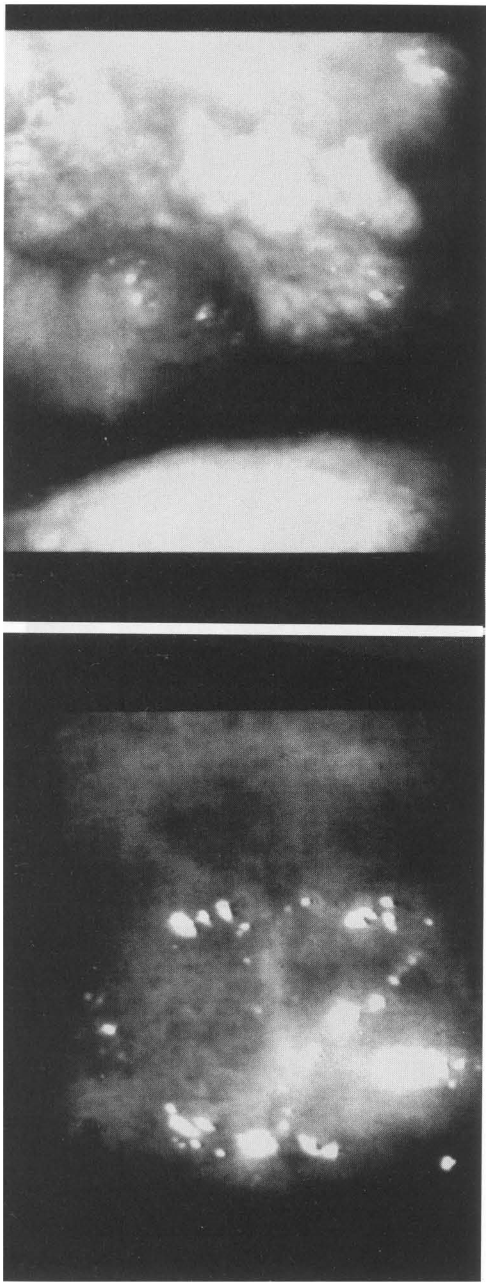

FIGURE 6 This patient (case 24) suffered from T3 pharyngeal cancer forming an extensive elevated tumor in the soft palate. Considering the advanced age of the patient (86 years old), PDT with a pulsed wave generated by an excimer dye laser (4 mJ, 30 Hz, 400 J/cm$^2$) was selected. Top, pretreatment view; bottom, 10 months after PDT.

### DISCUSSION

Radiotherapy is an established treatment for cancer of the head and neck and is used extensively in clinical practice. Multidisciplinary therapy including chemotherapy and surgery has improved treatment outcome. PDT has been reported to be effective as local therapy for early stage cancers [14,15]. Kato *et al.* [14] and Hayata *et al.* [16] have reported many clinical cases of lung cancer on the basis of fundamental research. Therefore, we attempted to use PDT for the treatment of cancers of the head and neck. Although HpD itself has no toxicity, photosensitization is an inevitable complication, and there also have been reports of recurrence after a CR was obtained by PDT [17]. We found that the longest disease-free survival in early stage laryngeal carcinoma by PDT alone was 67 months. In our study, cancer recurred in 1 patient treated only by PDT after having been in CR for 21 months. However, other patients with lingual or oral cancer and laryngeal carcinoma attained and maintained CR. The energy delivered by the argon dye laser ranged from 300 to 1056 J/cm$^2$. However, irradiation at an energy level of 300 J/cm$^2$ was not sufficient for local control; thus additional laser irradiation was performed in such patients. The energy of the excimer dye laser ranged from 100 to 650 J/cm$^2$, and local control was obtained in 11 of 13 patients. Furthermore, in 2 patients with T3 disease, the tumor disappeared and did not recur after irradiation of 200 to 400 J/cm$^2$ using an excimer dye laser. It was initially suspected that the excimer dye laser might not be effective in large advanced cancers; however, our results show that this is not the case. Moreover, because the excimer dye laser penetrates as deeply as 10 mm or more [18], it may be useful in patients who refuse surgical resection or whose general condition contraindicates surgery. The fact that a CR was obtained in 9 of 10 patients with T1 cancer in the larynx (including 1 patient with secondary PDT), and the single other patient, in which a PR had been obtained, refused another PDT session, suggests that PDT is comparable with conventional treatments in terms of therapeutic effect.

Normal mucosa showed mild inflammation and swelling immediately after PDT, the reaction reaching a peak after 24 hr. Tumor tissue showed a more intense reaction, forming fur and a crust, and some patients with laryngeal carcinoma developed dyspnea due to edema. Local reactions were especially intense in the treated area in patients with early stage cancer in whom maximum energy doses were given or areas previously treated by another modality or PDT. Some patients required daily endoscopic lavage, endoscopic removal of secretions, or nebulizer-aided expectoration. Although the patients with oral cancer manifested severe edema in the oral cavity, none had difficulty ingesting food, except 1 patient who had to be fed via a nasogastric tube. Our animal experiments and clinical experience established that energy exposure should be 200 J/cm$^2$ per PDT session, because at this energy level the local reaction is not so marked as to require complicated management or long-term lavage. However, since the photoradiation must be continued for 20 min even using a pulsed wave with 4 mJ, 40 Hz/pulse, it is necessary to maintain local anesthesia for at least that time.

The most important advantage of PDT is its ability to target tumor selectively with minimal adverse effects on other organs or surrounding tissue. PDT can be completed in one or two sessions and can be repeated if necessary. Another merit is the relative lack of toxicity. HpD tends to be distributed in connective tissue surrounding vessels. Since HpD accumulates mainly in the cell membrane, cytoplasm, and nuclear membrane, but not in the cell nuclei, it has no mutagenicity or carcinogenicity; therefore, it can be given safely to patients with severe complications or elderly patients. Furthermore, PDT can be included in multidisciplinary therapy and surgery or radiotherapy can be performed after reduction of the tumor mass by PDT. Palliative therapy of advanced cancer and local treatment of recurrent cancer are also possible because PDT does not prevent the application of other types of treatment [19].

Several new laser generators have been developed recently, such as the relatively inexpensive diode laser and the tunable wavelength Nd-YAG OPO laser, and they should be available commercially in the not too distant future. On the other hand, it is necessary to develop more effective and oncotropic photosensitizers. In addition to the currently used hematoporphyrin derivative photosensitizer [20], several new photosensitizers are under development with a view to improving therapeutic effect. These new substances include chlorophylls, chlorides [ATX-S10 [21,22]], Chlorin-e6 [NPe6 [23,24]], phthalocyanines [aluminum phthalocyanine [25–27]], silicon phthalocyanine [28], and pheophorbides (PH-1126), all of which are excited by laser beams with longer wavelengths than the light used with HpD so that histopermeability might be improved.

### SUMMARY

This report concerns the clinical course of 24 patients with head and neck cancer who underwent PDT and describes the efficacy, safety, and long-term results, as well as improved PDT techniques under endoscopic observation. The most important advantage of PDT is its ability to target malignant tissue selectively without adverse effects on other organs and is safe enough to apply even in elderly patients without risk of carcinogenecity. There are some reports that with respect to histologic findings, a CR is most likely to be achieved in squamous cell carcinoma. For further progress in the clinical practice of PDT in head and neck cancer, it is necessary to develop a laser beam that can penetrate tissue more deeply. Although the number of patients was limited, the long-term results appear equal or superior to those of conventional procedures, suggesting that PDT may be an effective strategy to treat cancer of the head and neck.

### *References*

[1] VonTappeiner, H., Jesionek, A. (1903). Therapeutische Versuche mit fluoreszierenden Stoffen, *Munch Med Wochenschr,* **1**, 2042–2044.

[2] Dougherty, T. J., Kaufman, J. E., Goldfarb, A., *et al.* (1978). Photoradiation therapy for the treatment of malignant tumors, *Cancer Res,* **38**, 2628–2635.

[3] Hayata, Y., Kato, H., Konaka, C., *et al.* (1982). Hematoporphyrin derivative and laser photoradiation in the treatment of lung cancer, *Chest,* **8**, 269–277.

[4] Abramson, A. L., Waner, M., Brandsma, J. (1988). The clinical treatment of laryngeal papillomas with hematoporphyrin therapy, *Arch Otolaryngol Head Neck Surg,* **114**(7), 795–800.

[5] Schuller, D. E., McCaughan, J. S., Rock, R. P. (1985). Photodynamic therapy in head and neck cancer, *Arch Otolaryngol,* **111**, 351–355.

[6] Yoshida, T., Saeki, T., Ohashi, S., *et al.* (1995). Clinical study of photodynamic therapy for laryngeal cancer, *Nippon Jibiinkoka Gakkai Kaiho (J Otolaryngol Jpn),* **98**, 795–804 [in Japanese].

[7] Lipson, R. L., Baldes, E. J. (1960). The photodynamic properties of a particular hematoporphyrin derivative, *Arch Dermatol,* **82**, 508–516.

[8] Kato, H., Horai, T., Furuse, K., *et al.* (1993). Photodynamic therapy for cancers; a clinical trial of porfimer sodium in Japan, *Jpn J Cancer Res,* **84**, 1209–1214.

[9] Bugelski, P. J., Porter, C. W., Dougherty, T. J. (1981). Autoradiographic distribution of hematoporphyrin derivative in normal and tumor tissue of the mouse, *Cancer Res,* **41**, 4606–4612.

[10] Kato, H., Konaka, C., Kawate, N., *et al.* (1986). Five-year disease-free survival of a lung cancer patient treated only by photodynamic therapy, *Chest,* **90**, 768–770.

[11] Yamamoto, H., Kato, H., Okunaka, T., *et al.* (1991). Photodynamic therapy with the excimer dye laser in the treatment of respiratory tract malignancies, *Lasers Life Sci,* **4**, 125–133.

[12] Kato, H., Konaka, C., Kinoshita, K., *et al.* (1990). Laser endoscopy with photodynamic therapy in the respiratory tract, *Gann Monogr Cancer Res,* **37**, 139–152.

[13] Dougherty, T. J., Cooper, M. T., Mang, T. S. (1990). Cutaneous phototoxic occurrences in patients receiving Photofrin, *Lasers Surg Med,* **10**, 485–488.

[14] Kato, H., Aizawa, K., Shinohara, H. (1986). Photodynamic diagnosis and therapy using laser, *Riza Kenkyu,* **14**, 208–218 [in Japanese].

[15] Kato, H., Konaka, C., Kawate, N., *et al.* (1987). Qualitative improvement of surgical treatment for cancer using laser equipment: surgical technique after photodynamic therapy, *Gann to Kagaku Ryoho (Jpn J Cancer Chemother),* **14**, 1468–1476 [in Japanese].

[16] Hayata, Y., Kato, H., Noguchi, M., *et al.* (1987). Photodynamic diagnosis and therapy for cancer using excimer dye laser, *BME,* **1**, 532–535.

[17] Wenig, B. L., Kurtzman, D. M., Grossweiner, L. I., *et al.* (1990). Photodynamic therapy in the treatment of squamous cell carcinoma of the head and neck, *Arch Otolaryngol Head Neck Surg,* **116**, 1267–1270.

[18] Sakai, H., Aizawa, K. (1987). Photoradiation therapy (PRT)—PDT by excimer laser, *Nippon Rinsho (Jpn J Clin Med),* **45**, 794–799 [in Japanese].

[19] Okunaka, T., Kato, H., Conaka, (Konaka) C., *et al.* (1990). Photodynamic therapy of esophageal carcinoma, *Surg Endosc,* **4**, 150–153.

[20] Chan, W. S., Svensen, R., Phillips, D., *et al.* (1986). Cell uptake, distribution and response to aluminium chloro sulphonated phthalocyanine, a potential anti-tumour photosensitizer, *Br J Cancer,* **53**, 255–263.

[21] Nakajima, S., Sakata, I., Takemura, T., *et al.* (1992). Tumor localizating and photosensitization of photochlorin ATX-S10. In: Spinelli P, Dal Fante M, Marchesini R, eds. Photodynamic therapy and biomedical lasers (Excerpta Medica International Congress Series no. 1011), *Amsterdam: Elsevier Scientific Publishers, Amsterdam,* 531–534.

[22] Haghighat, S., Castro, D. J., Lufkin, R. B., *et al.* (1992). Laser dyes for experimental phototherapy of human cancer; comparison of three rhodamines, *Laryngoscope,* **102**, 81–87.

[23] Roberts, W. G., Shiau, F. Y., Nelson, J. S., *et al.* (1988). In vitro characterization of monoaspartyl chlorin e6 and diaspartyl chlorine e6 for photodynamic therapy, *J Natl Cancer Inst,* **80**, 330–336.
[24] Dougherty T. J. (1993). Photodynamic therapy, *Photochem Photobiol,* **58**, 895–900.
[25] Tralau C. J., MacRobert A. J., (1987). Coleridge-Smith P.D., *et al.* Photodynamic therapy with phthalocyanine sensitisation: quantitative studies in a transplantable rat fibrosarcoma, *Br J Cancer,* **55**, 389–395.
[26] Rousseau J., Ali H., Lamoureux G., *et al.* (1985). Synthesis, tissue distribution and tumor uptake of $^{99m}$Tc- and $^{67}$Ga-tetra-sulfophthalocyanine, *Int J Appl Radiat Isotop,* **36**, 709–716.
[27] Rosenthal I. (1991). Phthalocyanines as photodynamic sensitizers, *Photochem Photobiol,* **53**, 859–870.
[28] Oleinick N. L., Antunez A. R., Clay M. E., *et al.* (1993). New phthalocyanine photosensitizers for photodynamic therapy, *Photochem Photobiol,* **57**, 242–247.

